# Osteoarticular Infection by Mycobacterium bovis Following Intravesical Bacillus Calmette-Guérin (BCG) Therapy: A Rare Case of Septic Arthritis and Osteomyelitis in an Immunocompromised Host

**DOI:** 10.7759/cureus.102629

**Published:** 2026-01-30

**Authors:** Margarida Santos, Ana Santos e Silva, Pedro Laranjo, Filipe Dias, Alexey Shigaev

**Affiliations:** 1 Internal Medicine, Unidade Local de Saúde do Litoral Alentejano, Santiago do Cacém, PRT

**Keywords:** intravesical bacillus calmette–guérin (bcg) therapy, mycobacterium bovis infection, osteoarticular infection, polyarthralgia, septic arthritis

## Abstract

Osteoarticular infections caused by *Mycobacterium bovis* are rare complications associated with intravesical Bacillus Calmette-Guérin (BCG) therapy and often cause subtle, nonspecific symptoms, which can delay the diagnosis and treatment.

We present the case of a 72-year-old male with a history of urothelial carcinoma treated with intravesical BCG therapy administered four years earlier, who developed persistent fever and progressive worsening polyarthralgia with a two-month duration prior to hospital admission. Imaging presented extensive joint and soft tissue inflammatory involvement. Early microbiological studies remained inconclusive, and the patient required several surgical debridements and broad-spectrum antimicrobial therapy empirically, later escalated to include antimycobacterial coverage. Definitive identification of a *Mycobacterium bovis *was obtained through genomic sequencing of synovial and bone specimens. A nine-month course of isoniazid, rifampicin, and ethambutol was initiated, leading to progressive clinical improvement.

Bacillus Calmette-Guérin-related osteoarticular infections are challenging conditions due to their rarity, insidious course, and usually the absence of microbiological confirmation by standard methods, especially in immunocompromised patients. Early suspicion, timely recognition, use of targeted microbiological tests, and a multidisciplinary approach are essential to achieve favorable outcomes.

## Introduction

Intravesical Bacillus Calmette-Guérin (BCG) therapy is a well-established treatment for non-muscle invasive urothelial carcinoma, with proven efficacy in reducing tumor recurrence and delaying disease progression [1,2]. While generally well tolerated, BCG - an attenuated live strain of *Mycobacterium bovis *- can cause infectious complications in less than 5% of cases, most commonly presenting as mild local infections such as cystitis, prostatitis, or immune-mediated reactive arthritis. Systemic osteoarticular infections such as osteomyelitis or septic arthritis have also been reported [3,4]. These complications are rare and may appear months or even years after the last instillation, making early diagnosis particularly challenging [5,6].

Osteoarticular tuberculosis (TB) is a rare but clinically significant form of extrapulmonary TB, accounting for 1-3% of all TB cases and 10-15% of extrapulmonary presentations [7-10]. It is most frequently caused by *Mycobacterium tuberculosis *(classical form), but *Mycobacterium bovis *infections, including iatrogenic cases following BCG instillation, have been described in the literature [3,4]. Weight-bearing joints such as the spine, hip, and knee are typically affected [9], though atypical presentations, including prosthetic joint infection, upper limb involvement, and disseminated disease [10-12], have been described, particularly in immunocompromised patients [7].

The diagnosis of BCG-related osteoarticular infection is difficult and often delayed due to its subtle or nonspecific symptoms [8], frequently unremarkable early imaging findings, and the frequent negativity of standard microbiological cultures [5,8]. As a result, diagnosis often requires a high index of suspicion and access to advanced microbiological diagnostic techniques and treatment demands prolonged combined antimycobacterial therapy and, in some cases, surgical intervention [5,13].

This case is noteworthy for its unusual presentation, combining progressive polyarticular involvement, long-term immunosuppression, repeated negative standard cultures, and a definitive diagnosis confirmed by genomic sequencing. It highlights the importance of recognizing late-onset BCG-related infection in immunocompromised patients with compatible clinical presentations and supports the need for advanced diagnostics and multidisciplinary management.

## Case presentation

A 72-year-old male was admitted to the emergency room with prolonged fever lasting over two months, predominantly vespertine in pattern and associated with chills, accompanied by progressively worsening polyarthralgia of the upper limbs, including shoulders, elbows, and wrists, sparing the fingers, with symptoms more pronounced in the left shoulder.

His clinical history included severe pulmonary emphysema (related to longstanding tobacco use, with a calculated load of 55 pack-years), with a previous episode of spontaneous pneumothorax, surgically treated with video-assisted thoracoscopic resection of the right upper and lower lobes. Additional comorbidities included heart failure with reduced ejection fraction and an implantable cardioverter-defibrillator (ICD) for primary prevention, stage 3 chronic kidney disease, arterial hypertension, and high-grade urothelial carcinoma previously managed with transurethral resections and intravesical immunotherapy with Bacillus Calmette-Guérin (BCG) between 2019 and 2020.

Additionally, he had an undifferentiated connective tissue disease with a scleroderma-like phenotype and had been receiving chronic immunosuppressive therapy with prednisolone (10 mg daily) and methotrexate (15 mg once weekly) for approximately one year. In the month preceding hospital admission, the patient also underwent periarticular injections of triamcinolone hexacetonide in both elbows for pain relief.

On admission, the laboratory tests revealed an elevated C-reactive protein (CRP) of 19 mg/dL, while other inflammatory markers (including leukocytes) were within normal levels. Initial imaging studies revealed significant articular inflammation: musculoskeletal ultrasound of the elbows showed “marked olecranon bursitis with joint effusion (grade III on the right, grade II on the left) associated with synovial hypertrophy”.

**Figure 1:**
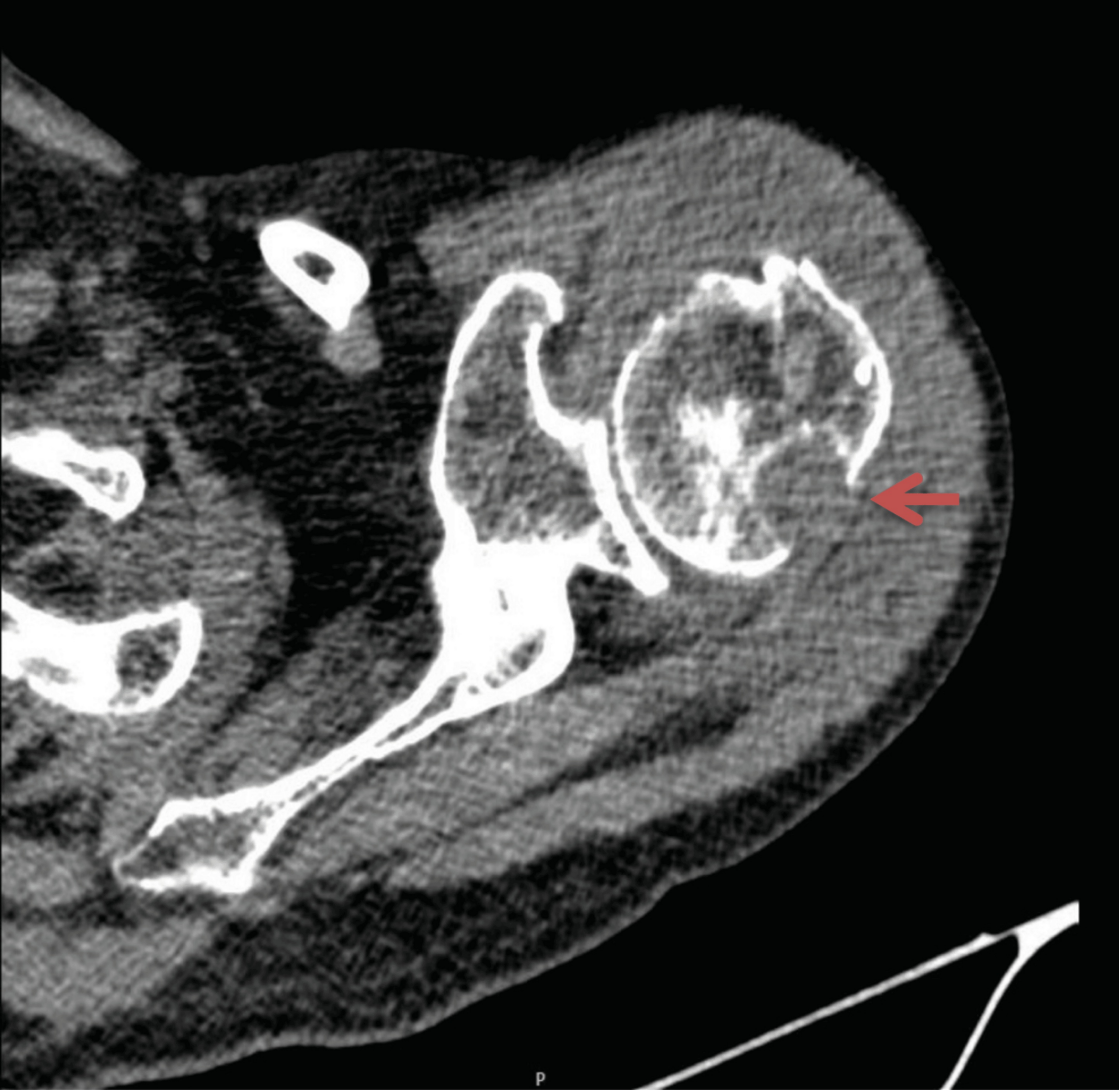
Right Shoulder CT scan demonstrating marked inflammatory changes Red arrow: Posterior aspect of humeral head with substantial erosion

A computed tomography (CT) scan of the left shoulder showed “glenohumeral joint effusion with synovitis, thickening and protrusion of the subacromial-subdeltoid bursa, and osseous erosions in the posterolateral region of the humeral head and acromion with sclerotic margins”, findings consistent with an active destructive inflammatory process and supporting the suspicion of osteoarticular infection (Figure 1). No signs of neoplastic disease were observed on additional imaging studies (thoraco-abdomino-pelvic and cranial CT scans).

A thorough investigation for common pathogens was conducted (blood and urine cultures, urinary antigens for *Legionella pneumophila *and *Streptococcus pneumonia*, screening for infectious diseases like HIV and hepatitis, and methicillin-resistant *Staphylococcus aureus* (MRSA)); all tests returned negative. 

Empirical antibiotic therapy was initiated with ceftriaxone and flucloxacillin, targeting the most frequent bacterial pathogens, given the clinical suspicion of a bacterial osteoarticular infection in the setting of prolonged fever, elevated inflammatory markers, and imaging findings suggestive of septic arthritis and osteomyelitis.

The analytical evolution is summarized in Table 1. 

**Table 1:** Progression of laboratory markers from initial presentation to discharge D0 represents the day of hospital admission; subsequent time points (D6, D12, etc.) correspond to days since admission. D37 corresponds to the start of the hospital-at-home program and D96 to the day of discharge. The table illustrates persistent inflammatory activity (C-reactive protein elevation), development of anemia and thrombocytopenia, and transient hepatic changes associated with antimicrobial therapy. These patterns support a prolonged inflammatory/infectious course and drug-related toxicity. TPO corresponds to AST (aspartate aminotransferase), and TGP corresponds to ALT (alanine aminotransferase).

Parameter	Reference values	D0 (Admission)	D6	D12	D16	D23	D28	D33	D37 (Hospital-at-home program)	D43	D52	D71	D82	D92	D96 (Discharge)
Haemoglobin (g/dL)	11.7-15.5	12.1	9.5	9.1	9.7	8.1	8.0	7.0	7.7	9.6	11.4	12.6	12.6	12.9	13.7
Leucocytes (x10^3^/uL)	4.0-11.0	9.1	6.7	6.3	5.5	3.6	3.3	2.9	6.1	5.0	3.6	6.7	6.5	6.9	7.7
Neutrophils (x10^3^/uL)	1.6-8.3	7.3	5.21	4.79	3.94	2.0	2.24	1.91	4.34	3.41	2.74	4.97	4.4	3.81	4.42
Platelets (x10^3^/uL)	150-400	321	313	334	271	176	128	83	101	194	86	275	219	266	305
Activated partial thromboplastin time (aPTT) (sec)	23.8-35.8	34.5	29.1	36.6	36.2	35.6	-	-	-	-	-	-	-	34.7	-
Urea (mg/dL)	< 43	63	49	58	60	53	49	44	-	77	89	60	74	80	99
Creatinine /mg/dL)	0.7-1.1	1.8	1.4	1.3	1.3	1.7	1.9	1.6	-	1.5	2.1	1.2	1.3	1.7	1.5
Sodium (mmol/L)	136-146	135	134	133	134	138	141	142	-	139	140	139	138	139	138
Potassium (mmol/L)	3.5-5.1	4.8	4.9	4.7	4.2	4.1	4.2	4.1	-	4.5	4.2	3.9	4.2	4.5	4.5
TPO (UI/L)	0-35	29	60	52	30	-	19	14	-	47	45	38	21	35	43
TGP (UI/L)	0-35	33	40	53	38	-	21	14	-	87	77	36	23	22	51
C-reactive protein (mg/dL)	< 0.5	19.09	29.31	9.96	4.62	6.70	3.40	1.40	-	6.20	10	3.10	3.50	3.80	3.20
Procalcitonin (ng/mL)	< 0.5	-	0.70	0.22	0.19	0.17	-	0.18	-	-	-	-	-	0.07	-

On the sixth day of hospitalization, an exploratory surgical procedure of the left shoulder was performed by the orthopedic team. Copious purulent exudate was found, with septic collections in the anterior and posterior deltoid compartments, involving the subacromial space and rotator cuff, along with bone destruction in the posterolateral region of the humeral head extending into the humeral diaphysis. Extensive debridement and irrigation with high volumes of diluted rifampicin and saline solution were performed.

Samples of synovial fluid, muscle, and tendinous tissue from the deltoid, rotator cuff tendons, and bone fragments were sent for bacterial, fungal, and mycobacterial cultures, all of which returned negative by conventional microbiological methods.

Following surgery, intravenous vancomycin was initiated in light of the intraoperative findings of purulent collections and ongoing suspicion of extensive infection, aiming to ensure adequate coverage of Gram-positive organisms, including methicillin-resistant *Staphylococcus aureus*. Vancomycin was later replaced by linezolid, maintaining equivalent antimicrobial coverage in the context of a complex osteoarticular infection.

Continuing the etiologic study, a transthoracic echocardiogram was performed to rule out endocarditis (no vegetations were found), and the cardiology team deemed the transesophageal study unnecessary. MRI of the upper limb was not performed due to an incompatible dental prosthesis.

**Figure 2:**
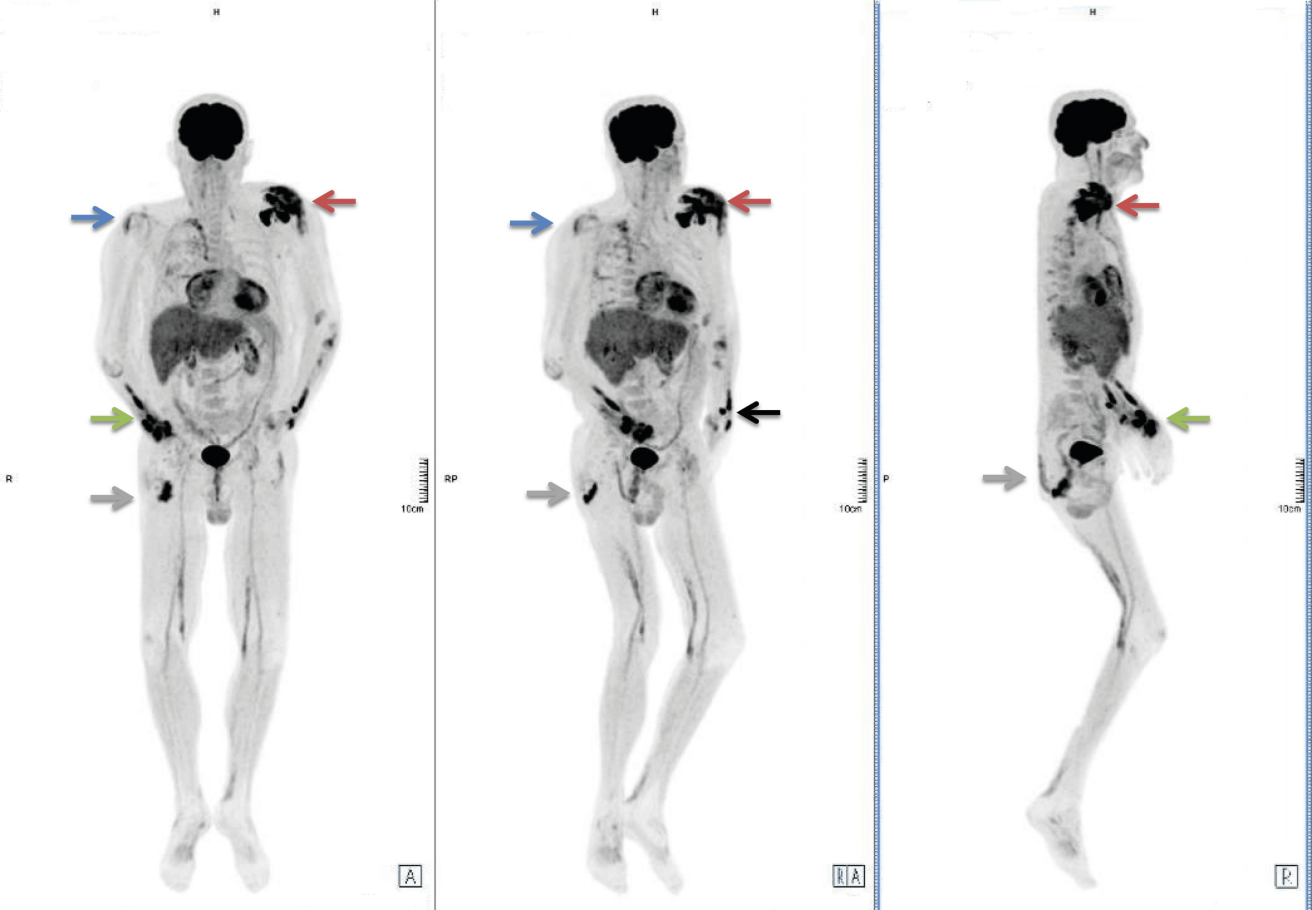
PET scan revealing multiple hypermetabolic regions consistent with active infectious disease Red arrow: left shoulder hypermetabolism; Blue arrow: right shoulder involvement; Green arrow: right wrist and soft tissue involvement; Grey arrow: right gluteal area involvement; Black arrow: left wrist and soft tissue involvement A: anatomical position, anterior view; RA: anterior view with slight rightward rotation; R: right lateral view

A positron emission tomography (PET) scan was performed, which revealed hypermetabolic foci in the left shoulder, wrists, and soft tissues of the forearms, right gluteal region, as well as in the right shoulder, consistent with multifocal inflammatory or infectious involvement, despite repeatedly negative results from conventional microbiological testing (Figure 2).

On the sixteenth day, new inflammatory signs developed in the right wrist (with swelling and functional impairment). CT scan of the wrist showed significant distention of the ulnar bursa and marked synovitis of the radiocarpal joint, needing a second surgical debridement. Further tissue samples were collected for culture, again yielding negative culture results.

Given the persistence of the infectious process without pathogen identification, despite repeated sampling and broad-spectrum antibiotic therapy, suspicion arose for an atypical organism. This concern was further supported by the patient’s history of intravesical BCG therapy and the subacute, progressive clinical course. Empirical antimycobacterial therapy was therefore initiated with tigecycline (50 mg every 12 hours), in combination with rifampicin (600 mg once daily) and ethambutol (25 mg/kg daily), while awaiting targeted microbiological results.

Results returned positive for *Mycobacterium *complex, and isoniazid was added to the treatment regimen.

Subsequently, genomic sequencing confirmed the *Mycobacterium bovis *BCG strain in both bone and synovial tissue samples. The antimicrobial regimen was adjusted to rifampicin, isoniazid, and ethambutol to be maintained for a total duration of nine months. Due to drug-induced hepatotoxicity, the regimen was briefly interrupted and reintroduced with close monitoring.

**Figure 3:**
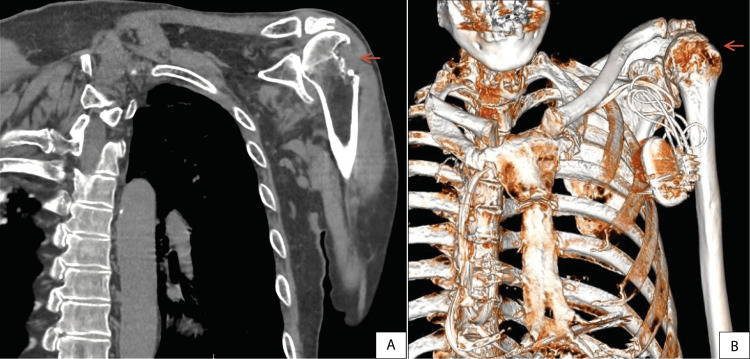
Follow-up CT scan and 3D reconstruction, showing residual structural damage in the right shoulder, illustrating long-term sequelae despite microbiological resolution. A: CT scan (coronal view); B: three-dimensional (3D) CT scan reconstruction (volume rendering technique) of the left shoulder; Red arrow: demonstrates residual bone destruction following *Mycobacterium bovis*-related osteoarticular infection

After 95 days of hospitalization (the last 56 in a domiciliary hospitalization program), the patient was discharged with a multidisciplinary follow-up, including internal medicine, pulmonology, rheumatology, physical and rehabilitation medicine, and orthopaedics. He showed gradual clinical improvement with resolution of systemic symptoms, although reduced mobility persisted. One year later, a follow-up CT scan demonstrated evident structural sequelae in the right shoulder (Figure 3).

Overall, this case highlights a late-onset clinical scenario characterized by sustained inflammatory activity, progressively evolving polyarticular involvement, and consistently negative conventional microbiological studies occurring in an immunocompromised host following intravesical BCG therapy.

## Discussion

Bacillus Calmette-Guérin (BCG) is an attenuated live strain of *Mycobacterium bovis*, initially used as an anti-tuberculosis vaccine [3,4], and later used as immunotherapy in the treatment of urothelial carcinoma [1,2]. Although generally safe, this therapy is associated with a spectrum of potential complications that may present months or years after treatment, ranging from severe cystitis and ulcerations to systemic dissemination, such as sepsis or osteomyelitis [9]. The reported cases describe variable latency periods, from a few weeks to several years after the end of the therapy cycle [5, 6], with the most commonly affected anatomical structures being the thoracic and lumbar vertebrae and prosthetic joints (mostly the hip) [9-12].

In this context, the present case expands the clinical spectrum of BCG-related disease by illustrating a delayed, progressive, and polyarticular presentation, emphasizing that late manifestations are not only possible but clinically relevant, especially in immunocompromised hosts.

A retrospective analysis of longitudinal laboratory trends (presented in Table 1) provides important diagnostic and management insights. The persistent elevation of C-reactive protein over several weeks, despite broad-spectrum antimicrobial therapy and surgical source control, supported the presence of an ongoing active infectious process rather than a self-limited inflammatory or immune-mediated process. The early development of anemia and thrombocytopenia was likely multifactorial, reflecting sustained inflammatory burden and treatment-related effects, and reinforced the need for close hematologic monitoring during prolonged therapy. Subsequent hepatic parameter elevation following initiation of antimycobacterial treatment was consistent with drug-induced hepatotoxicity and improved after dose adjustment. Together, these data give a better understanding of the temporal evolution and clinical complexity of the disease course.

The diagnosis of osteoarticular tuberculosis secondary to intravesical BCG instillation represents a significant clinical challenge and requires a high degree of suspicion, since the symptoms are often insidious and nonspecific, the initial imaging findings can be unremarkable, and microbiological isolation is frequently unsuccessful, requiring more specific and sometimes time-consuming tests [1, 5]. In this case, the persistent inflammation despite appropriate empirical antibiotics, the multifocal involvement on imaging, and repeatedly negative conventional cultures constitute diagnostic red flags. 

An essential diagnostic consideration in patients exposed to intravesical BCG is the distinction between mycobacterial infection and immune-mediated reactive arthritis, which represents a sterile inflammatory response to BCG and follows a more benign course with response to anti-inflammatory therapy [14]. In contrast, this case demonstrated active infection by *Mycobacterium bovis,* confirmed by genomic sequencing with direct involvement of bone and synovial tissues, excluding the reactive etiology. The distinction has major clinical implications, as it directly influences treatment strategy and prognosis [14].

In this patient, the coexistence of chronic immunosuppression (secondary to autoimmune disease, under corticosteroid therapy) may have favored hematogenous dissemination of the microorganism following intravesical BCG instillation, leading to the observed multiple joint involvement [11]. Furthermore, corticosteroid infiltrations (given one month earlier) are independently associated with opportunistic infections by promoting local colonization [12, 15, 16], initially complicating the diagnostic process. Although infection screening prior to corticosteroid injections in immunosuppressed patients is not standard practice, it may be considered in selected cases, particularly when there are unexplained systemic symptoms.

The treatment is similar to that for other *Mycobacterium* species, with the exception of pyrazinamide, as *Mycobacterium bovis* exhibits intrinsic resistance to this drug. The recommended treatment duration typically ranges from 9 to 12 months, depending on the extent and severity of the disease [12, 13]. More severe cases, such as extensive osteoarticular disease or periarticular abscesses, often require surgical treatment, as described in previous reports [4]. Overall, outcomes are generally favorable when timely combined medical and surgical management is instituted [3].

## Conclusions

It is important to consider *Mycobacterium bovis* osteoarticular infection in the differential diagnosis of an immunocompromised patient with a history of previous intravesical BCG instillation, particularly when presenting with chronic or progressive joint involvement.

This case illustrates how a delayed clinical presentation, occurring several years after BCG instillation, together with persistent inflammatory activity, polyarticular involvement, and repeatedly negative conventional microbiological studies, may obscure early diagnosis and contribute to diagnostic delay. In such settings, early clinical suspicion and timely use of targeted microbiological and molecular diagnostic techniques are essential to establish the diagnosis and guide appropriate therapy.

Favorable clinical outcomes may be achieved through a multidisciplinary approach, combining prolonged antimycobacterial treatment with surgical intervention for source control when indicated.
